# Fostering Happiness: A Pilot Study on Enhancing Psychological Well-Being of Nursing Students

**DOI:** 10.3390/nursrep16090318

**Published:** 2026-09-04

**Authors:** Erica Blumenstock, Debra Penrod, Heather Brown

**Affiliations:** School of Health Sciences-Nursing, Southern Illinois University Carbondale, Carbondale, IL 62901, USA; debra.penrod@siu.edu (D.P.); heather.n.brown@siu.edu (H.B.)

**Keywords:** nursing students, nursing education, stress management, resilience, mindfulness, mental health, well-being, psychological well-being, complementary therapies, breathwork

## Abstract

**Background:** In the wake of the COVID-19 pandemic, nursing students and educators are navigating an increasingly complex academic environment. The rigorous demands of nursing education, combined with diminished academic preparedness and growing mental health concerns, have heightened the need for effective wellness interventions. The SKY Happiness Retreat©, an evidence-based program incorporating breathwork, meditation, and mindfulness practices, offers a potential strategy to enhance student resilience and reduce stress. **Purpose:** This pilot study examined the impact of integrating the SKY Happiness Retreat© into a Bachelor of Science in Nursing (BSN) program. This study evaluated whether participation reduced perceived stress and improved students’ ability to manage stressful situations. **Methods:** A quasi-experimental pretest–post-test design was used with junior-level BSN students recruited through convenience sampling at a rural university. Participants completed a structured three-day retreat focused on breathwork, meditation, and mindfulness. Outcomes were measured using the Mood and Anxiety Symptom Questionnaire (Mini-MASQ), the Perceived Stress Scale (PSS), and the Brief Resilience Scale (BRS). **Results:** Perceived stress significantly decreased following the intervention (PSS: pre M = 18.50, SD = 4.583; post M = 17.30, SD = 5.841; *t*(43) = 2.072, *p* = 0.022). General distress also significantly improved (Mini-MASQ: pre M = 15.51, SD = 6.535; post M = 12.82, SD = 5.118; *t*(44) = 3.941, *p* < 0.001). No significant changes were observed in anxious arousal or anhedonic depression. BRS scores demonstrated a modest increase in resilience. **Conclusions:** The SKY Happiness Retreat© shows promise as an experiential learning strategy within undergraduate nursing education. Findings suggest participation was associated with reductions in perceived stress and general distress while promoting resilience. Integrating evidence-based wellness programs into prelicensure nursing curricula may better prepare students to manage academic and professional stress. Larger, longitudinal studies are needed to evaluate long-term effectiveness.

## 1. Introduction

Students and nurse educators face ongoing challenges related to academic readiness in higher education. National Assessment of Educational Progress data indicate declines in students’ academic performance, raising concerns that some students may enter college less prepared for the rigor of nursing coursework [1]. This readiness gap may require nurse educators to provide additional academic support while maintaining the standards necessary to prepare students for complex clinical practice. The rigor of a nursing program and a lack of academic readiness may create an environment where students experience a decline in mental well-being. Nurse educators are left struggling to balance preparing students for practice in a challenging healthcare environment while promoting their mental health.

*The Future of Nursing 2020–2030* report encourages nursing programs to identify and address barriers to student success. Nursing programs are encouraged to implement targeted interventions that address these barriers and promote student retention. In addition, the report discusses how the lack of well-being negatively impacts individual nurses, patients, and society and calls on the organizations that support nurses to promote well-being [2].

Educators face many challenges when identifying clinical experiences for students in specialty areas such as mental health. These challenges are exacerbated for programs located in rural areas, particularly in clinical specialties such as mental health. Innovation is needed to identify experiential learning strategies that enhance student well-being and ensure their success in future work environments.

The SKY Happiness Retreat© is a research-based program for students, faculty, or staff. It provides skills to enhance well-being and resilience [3]. The retreat was implemented as an innovative clinical approach for nursing students at a rural university to teach methods for responding to stressful situations and improving well-being.

### 1.1. Literature Review

Nursing students encounter overlapping academic, clinical, and emotional demands that can affect mental health, academic performance, retention, and readiness for practice. These concerns warrant preventive approaches that strengthen coping before students enter high-stakes clinical environments. This review summarizes evidence on stress, resilience, mindfulness, and structured well-being interventions relevant to prelicensure nursing education.

### 1.2. Stress, Well-Being, and Resilience in Nursing Students

Stress is common across nursing education and professional practice. Among practicing nurses, occupational stressors include workload, exposure to death and dying, inadequate emotional preparation, and insufficient support. Babapour et al. found that greater job stress was significantly associated with poorer quality of life (*r* = −0.44, *p* < 0.001) and reduced caring behaviors (*r* = −0.26, *p* < 0.001) [4]. During the transition from education to practice, job stress is also associated with less favorable patient-safety attitudes, whereas supportive practice environments can mitigate these effects [5]. Emotional intelligence (EI) and self-efficacy are pivotal in managing stress among nurses. Molero Jurado et al. [6] found that higher emotional intelligence was associated with lower perceived stress among nursing professionals. Self-efficacy was also identified as an important predictor of perceived stress, suggesting that nurses who are more confident in their ability to manage challenging situations may experience lower stress levels. The observed gender differences in emotional intelligence further suggest that stress-management interventions may benefit from considering individual characteristics and strengths. Nursing students experience similar pressures during academic and clinical training, including fear of errors and difficulty responding to emergencies [7,8]. Problem-solving and social support are associated with more adaptive coping, while avoidant strategies are associated with greater perceived stress [8].

Resilience may help students adapt to these demands. Higher self-efficacy and other personal resources have been associated with greater wellness and resilience [9], and resilience-building content may support students’ academic and professional development [10,11]. However, evidence supporting the effectiveness of resilience-focused educational interventions remains limited. In a 2024 systematic review and meta-analysis of six studies involving 472 nursing students, Ejaz et al. found no significant immediate pooled improvement in resilience in either quasi-experimental studies or randomized controlled trials. However, a significant improvement in resilience was observed at the one-month follow-up in the randomized controlled trials [12]. These findings suggest that resilience is multidimensional and may change gradually, supporting sustained, theory-informed programming rather than a single brief exposure.

### 1.3. Preventive Stress-Management Approaches

University students commonly report anxiety, depression, and stress related to academic demands, social adjustment, and life transitions, with consequences for sleep, relationships, and academic functioning [13]. Because nursing students face these general pressures in addition to clinical responsibilities, preventive interventions embedded in the curriculum may be more accessible than relying solely on services initiated after distress becomes severe.

Recent evidence supports the value of structured stress-management interventions for nursing students. Ji et al.’s 2024 systematic review and meta-analysis included 41 controlled studies, with 36 studies and 2715 participants contributing to the pooled analysis [14]. Interventions produced a moderate-to-large reduction in psychological stress (SMD = −0.66, 95% CI −0.79 to −0.52) and were also associated with reductions in systolic blood pressure, heart rate, and cortisol. Mind–body programs, on-site delivery, 9–12-week duration, and 15–30 sessions showed stronger effects, although limited follow-up and study heterogeneity restricted conclusions about long-term effectiveness [14].

Mindfulness-based interventions are among the most frequently studied preventive approaches. A meta-analysis of 10 randomized controlled trials found significant reductions in depression (SMD = −0.42), anxiety (SMD = −0.32), and stress (SMD = −0.50), together with increased mindfulness (SMD = 0.54), among nursing students [15]. However, only one included study reported follow-up data, emphasizing the need to evaluate whether benefits persist. Likewise, mindfulness-related skills such as self-compassion and psychological flexibility are associated with better mental health, but may not fully buffer the relationship between academic stress and anxiety or depression [16]. Thus, mindfulness may be most useful as one component of a broader preventive strategy.

The SKY Campus Happiness program© combines breath regulation, meditation, mindfulness, and social connection. In a randomized trial of university students, SKY© produced greater improvements in depression, stress, mental health, mindfulness, positive affect, and social connectedness than comparison interventions, with several benefits maintained at follow-up [17]. This combination is particularly relevant to nursing education because it targets perceived stress while also developing self-regulation and interpersonal connection. Nevertheless, evidence specific to prelicensure nursing students remains limited.

Collectively, the literature supports proactive, curriculum-based stress-management programming for nursing students, while also identifying important gaps. Meta-analytic evidence demonstrates meaningful short-term benefits of mindfulness and broader stress-management interventions [14,15], whereas resilience-focused programs may require time and repeated practice before measurable effects emerge [12]. These findings provide a rationale for evaluating SKY© as an experiential wellness intervention in prelicensure nursing education and for examining multiple outcomes, including perceived stress, psychological distress, and resilience.

Although existing research supports the potential benefits of mindfulness and stress-management interventions for nursing students, less is known about intensive, retreat-based programs incorporated directly into prelicensure nursing curricula. Further research is needed to determine whether these programs can improve student well-being while also serving as feasible experiential learning opportunities within rural nursing education.

### 1.4. Purpose and Research Questions

Nursing students experience substantial academic and clinical stress, yet nursing programs, particularly those in rural settings, may have limited access to specialty mental health and structured wellness experiences. Although previous studies have examined mindfulness, resilience, and stress-management interventions among nursing and university students, limited research has evaluated an intensive, retreat-based wellness intervention embedded within a prelicensure mental health nursing course.

This pilot study addressed this gap by examining changes in perceived stress, resilience, and psychological responses to stressful situations among BSN students following participation in the SKY Happiness Retreat© as an experiential wellness intervention.

The following research questions guided the study:

1. Did nursing students experience decreased perceived stress after participating in the SKY Happiness Retreat©?
2. Did nursing students experience improvements in resilience and psychological responses to stressful situations after participating in the SKY Happiness Retreat©?

## 2. Materials and Methods

This study employed a quasi-experimental design with a pretest–post-test approach to collect quantitative data, aiming to provide insights into the impact of the SKY Happiness Retreat© on nursing students. Participants were junior nursing students enrolled in a Bachelor of Science in Nursing (BSN) program in rural Illinois. Clinical hours were awarded to students who participated in the retreat. Students who chose not to participate were offered optional activities to fulfill their clinical hours. A total of 45 students participated in the retreat. Quantitative data were collected via a structured online survey using validated scales to measure anxiety, stress, and resilience.

### 2.1. Ethical Considerations

This study was conducted in accordance with the ethical principles outlined in the Declaration of Helsinki. Participation was voluntary, and informed consent was obtained from each subject before their involvement. The participants were informed of the research’s purpose and any potential risks and benefits. We designed this study to avoid unnecessary physical or mental harm and made efforts to minimize risk. This study was reviewed and approved by the Institutional Review Board of Southern Illinois University Carbondale under the expedited category (Protocol #24077). Before participation, informed consent was obtained from all individuals, ensuring they understood their rights, including confidentiality and the ability to withdraw at any time. Neither organization was provided access to the research data or had any role in the study’s conduct, analysis, interpretation, or reporting. The authors are not affiliated with either organization or with the SKY Happiness Retreat© program and have no personal or financial relationships that could be perceived as influencing the research. All collected data are securely stored in a password-protected online system, and printed copies were shredded after use to protect data security and participant privacy. No external funding was received for this study.

### 2.2. Procedure

The SKY Happiness Retreat© is a structured well-being program that incorporates breathing techniques, meditation, and mindfulness practices. In this study, participants were recruited from junior-level nursing students enrolled in a baccalaureate-level nursing program. Upon providing informed consent, they completed an online pretest survey assessing baseline measures of well-being, stress, and mental health.

Participants completed the SKY Happiness Retreat©, an evidence-informed, experiential well-being program designed to enhance psychological resilience, emotional regulation, stress management, and social connectedness. The retreat integrates evidence-based practices, including controlled yogic breathing (Sudarshan Kriya Yoga), mindfulness meditation, gentle yoga, positive psychology principles, self-reflection, and facilitated group discussion. The intervention is grounded on the premise that the intentional regulation of breathing, combined with mindfulness practices and social connection, can improve autonomic regulation, emotional well-being, and resilience in response to stress [17].

The retreat was delivered over three consecutive days by certified SKY© instructors using a standardized curriculum. Instruction combined didactic content with experiential learning, allowing participants to actively practice and reflect upon each technique. Throughout the program, students were introduced to strategies for recognizing physiological and emotional responses to stress and guided in developing practical skills to respond more effectively to academic and personal challenges.

The retreat incorporated multiple complementary components designed to promote psychological well-being and resilience. Participants engaged in evidence-based breathing practices intended to regulate the autonomic nervous system, including Ujjayi (“Victorious Breath”), Bhastrika (“Bellows Breath”), Nadi Shodhan (alternate nostril breathing), and the Sudarshan Kriya breathing technique. Guided mindfulness and meditation sessions were integrated throughout the retreat to cultivate present-moment awareness, enhance attentional control, and foster emotional regulation. Gentle yoga and stretching exercises were included to increase mind–body awareness while reducing physical manifestations of stress. Interactive experiential activities emphasized gratitude, self-awareness, interpersonal communication, and positive psychology principles to strengthen resilience and promote psychological flourishing. The retreat also incorporated facilitated small-group discussions and structured reflective exercises that encouraged participants to recognize personal stressors, develop adaptive coping strategies, strengthen social connectedness, and explore personal values, meaning, and purpose.

Unlike interventions that solely focus on mindfulness or psychoeducation, the SKY Happiness Retreat© incorporates multiple complementary components, including breathwork, meditation, yoga, positive psychology, leadership development, and community-building activities. Previous randomized controlled research among university students has demonstrated that the SKY© program significantly improves perceived stress, depressive symptoms, mindfulness, positive affect, social connectedness, and overall mental health compared with control interventions, supporting its use as a comprehensive student well-being intervention [17]. However, its use as an intensive, curriculum-embedded experiential activity for prelicensure nursing students has received limited investigation, particularly within rural nursing programs.

After the retreat, participants completed an online post-test survey measuring changes in psychological and emotional well-being. Data were collected anonymously, and all responses were securely stored in a password-protected database for analysis. Data from participants who completed both the pre- and post-intervention assessments were included in the matched-pairs analyses. Statistical analyses were conducted using jamovi statistical software version 2.2.28. Paired-samples *t* tests were used to compare pre- and post-intervention scores on the Perceived Stress Scale, Brief Resilience Scale, and overall Mini-MASQ, as well as the Mini-MASQ general distress, anxious arousal, and anhedonic depression subscales. This test was selected because each analysis compared two continuous measurements obtained from the same participants before and after the intervention. Directional, one-tailed tests were used because the research questions specified anticipated decreases in perceived stress and adverse psychological responses and an increase in resilience following participation. Statistical significance was established at *p* < 0.05. Cohen’s *d* and 95% confidence intervals were reported to describe the magnitude and precision of the observed changes.

### 2.3. Sampling

An a priori power analysis was not conducted because this exploratory pilot study was designed to obtain preliminary estimates of preintervention-to-postintervention changes, effect sizes, and variability in perceived stress, psychological distress, and resilience rather than providing a definitive test of intervention efficacy. These preliminary estimates may inform outcome selection and sample-size calculations for future controlled studies. Recruitment emails were sent to all 91 eligible third-year BSN students enrolled in the participating cohort. Participation was entirely voluntary, and students could decline without penalty or effect on their course standing. The final sample consisted of 45 participants. The study was designed to assess feasibility and estimate preliminary pre–post changes rather than provide a definitive test of efficacy. Findings and effect-size estimates from this pilot study may inform sample-size calculations and the design of a future controlled study.

The study sample consisted primarily of junior-level nursing students, with the majority (75.6%) between the ages of 21 and 29 and the minority (22.2%) between 18 and 20. A small minority of participants (2.2%) were between 50 and 59 years of age.

Overall, 71.1%, 26.7%, and 2.2% of the participants were enrolled in a traditional nursing program, an accelerated program, and an MSN program, respectively. Regarding graduation timelines, 42.2% were expected to graduate in 2024, while 57.8% were projected to complete their program in 2025. The racial composition of the sample was predominantly White (82.2%), followed by Black or African American (13.3%) and Asian (4.4%). Most participants (86.7%) identified as female, 11.1% as male, and 2.2% as trans-masculine. In terms of sexual orientation, 82.2% identified as heterosexual, while 11.1% identified as bisexual, 4.4% as queer, and 2.2% as pansexual. Most participants (64.4%) were employed part-time, while 31.1% were not employed and not looking, and 4.4% were not employed but actively seeking work. Educational background varied, with 57.8% having completed college, 20% holding a high school diploma, 13.3% earning an associate degree, 6.7% a bachelor’s degree, and 2.2% a graduate degree.

### 2.4. Participant Flow and Missing Data

Recruitment emails were sent to 91 eligible junior-level BSN students. Participation was voluntary, and students could decline without penalty or any effect on their course standing. Of the eligible students, four students declined participation. An additional 43 students attended the retreat but did not complete the informed consent process or study questionnaires; therefore, they were not enrolled in the study. The final enrolled sample included 48 participants. Of these participants, 45 provided matched preintervention and postintervention Mini-MASQ data, while 44 provided matched preintervention and postintervention data for both the Perceived Stress Scale and Brief Resilience Scale. Postintervention demographic information was available for 42 participants. Thus, three participants were excluded from the Mini-MASQ analysis, four were excluded from the PSS and BRS analyses, and six did not provide demographic information. The specific reasons for incomplete assessments were not systematically documented.

### 2.5. Instruments/Measurements

To assess the SKY© retreat’s efficacy in reducing stress in nursing students, participants were asked to complete a series of questionnaires to measure stress symptoms before and after the retreat. The survey comprised questions from the following validated tools: the Mood and Anxiety Symptom Questionnaire (Mini-MASQ), Perceived Stress Scale (PSS), and the Brief Resilience Scale (BRS).

The mini-MASQ was utilized to assess participants’ mood and anxiety symptoms. The mini-MASQ is a shortened version (26 items) of the MASQ (62 items). The shortened version was found to have validity in measuring anxiety and depression symptoms [18]. The scale scores subjects in the following categories: general distress, anxious arousal, and anhedonic depression. Generalized distress is a term for the inability of people to cope or adapt to stressors or a failure to return to homeostasis [19]. Generalized distress occurs from prolonged exposure to acute and chronic stress that can accelerate aging, cognitive decline, and mortality if untreated. Anxious arousal is a type of anxiety (also considered somatic anxiety) that represents the immediate response when encountering a stressor. Symptoms of anxious arousal resemble fight-or-flight responses, with physiological symptoms of increased heart rate and shortness of breath [20]. Anxious arousal can be a healthy response to an immediate stressor but can be maladaptive when at rest or when the threat disappears. Anhedonic depression is characterized by a lack of enjoyment, motivation, and interest in the person’s environment [21]. People who are diagnosed with anhedonic depression are apathetic toward life experiences, regardless of positive or negative, and have difficulty making decisions. Physiological symptoms of this type of depression include lack of sleep, social withdrawal, and low energy and fatigue that can lead to more social isolation and suicidal thoughts [22].

The PSS assessed participants’ stress levels. This PSS demonstrated reliability and validity across three samples, two of which comprised college students in measuring perceived stress [23].

The BRS measures participants’ resilience or ability to recover from stress [24]. It was found that the scale was a reliable tool for assessing this ability.

## 3. Results and Discussion

Analyses were conducted separately for each outcome using participants with complete matched preintervention and postintervention data for the applicable measure. Missing responses were treated as missing and were not imputed. Consequently, the analytic sample size varied by outcome: 45 participants for the Mini-MASQ and its subscales and 44 participants for the PSS and BRS.

### 3.1. Psychological Distress

**Figure 1:**
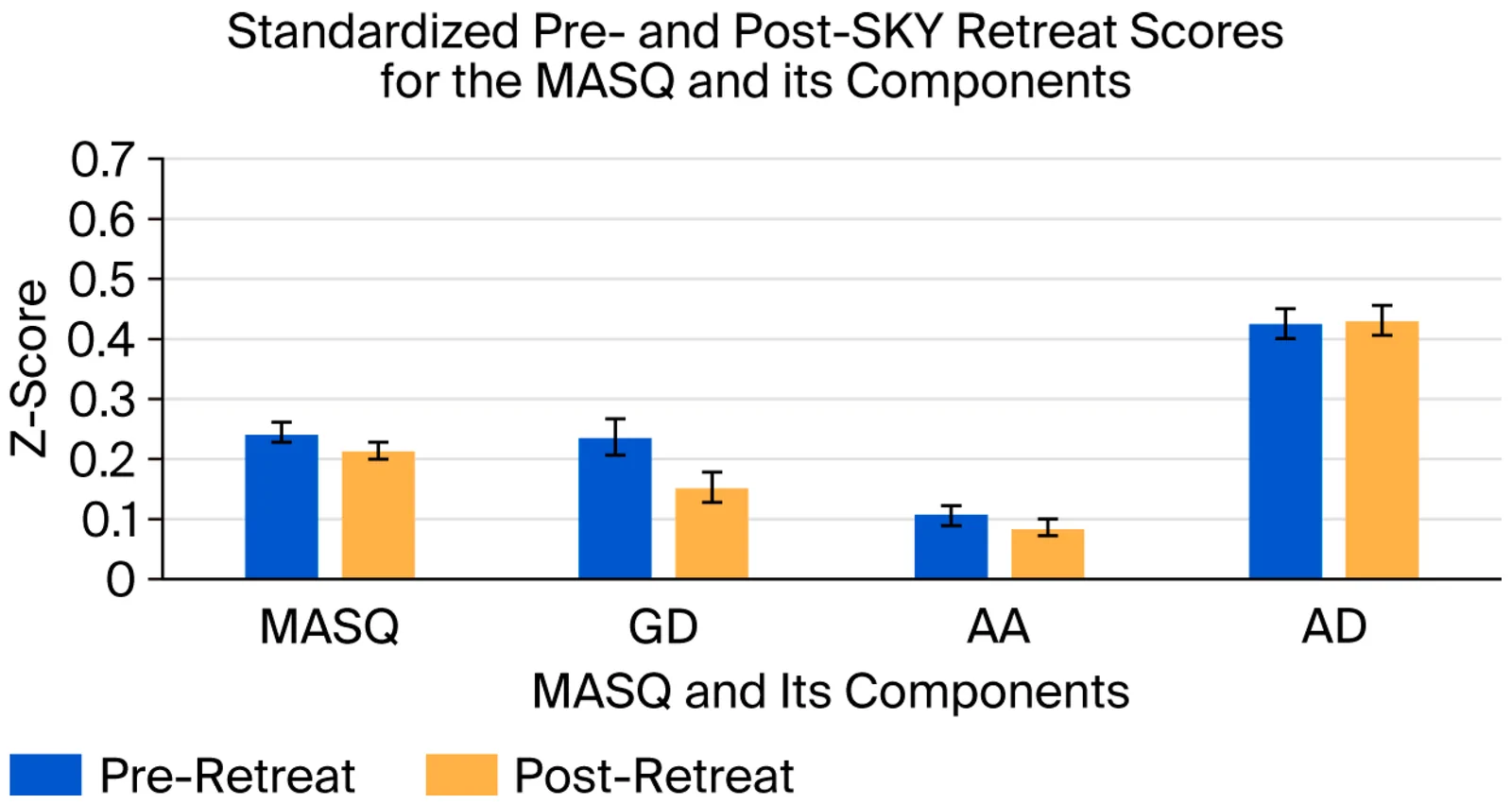
Pre- and post-SKY© retreat scores for the MASQ and its components.

Overall Mini-MASQ scores decreased significantly from preintervention (M = 51.24, SD = 12.277) to postintervention (M = 47.98, SD = 9.829), *t*(44) = 2.670, *p* = 0.005, 95% CI [0.801, 5.732], Cohen’s *d* = 0.398. Among the Mini-MASQ subscales, general distress decreased from preintervention (M = 15.51, SD = 6.535) to postintervention (M = 12.82, SD = 5.118), *t*(44) = 3.941, *p* < 0.001, 95% CI [1.314, 4.064], Cohen’s *d* = 0.588. No statistically significant changes were observed for anxious arousal, *t*(44) = 1.165, *p* = 0.125, 95% CI [−0.519, 1.941], or anhedonic depression, *t*(44) = −0.219, *p* = 0.414, 95% CI [−1.360, 1.093] (Figure 1).

The figure shows standardized z-scores for the MASQ and its components: general distress (GD), anxious arousal (AA), and anhedonic depression (AD). Error bars represent the standard error of the mean.

These findings suggest that participation in the retreat was associated primarily with improvement in general psychological distress rather than with changes across all mood and anxiety domains. This pattern is generally consistent with previous research on the SKY Campus Happiness© program among university students. Seppälä et al. reported improvements in perceived stress and psychological well-being, although the benefits were not uniform across every mental health outcome [16]. The present findings extend this evidence by demonstrating a similar pattern among prelicensure nursing students, who experience distinct academic and clinical stressors.

The intervention’s emphasis on controlled breathing, mindfulness meditation, and guided reflection may have provided students with immediately applicable strategies for regulating responses to everyday stress, consistent with evidence supporting stress-management and mindfulness-based interventions among nursing students [14,15]. However, anxious arousal and anhedonic depression represent distinct and potentially complex symptom domains [20,21]. Therefore, the absence of significant changes in these outcomes may reflect differences in how these symptoms respond to a brief intervention, although the present study cannot determine the reason for this finding. Future research should examine whether longer interventions, repeated practice, or extended follow-up periods produce changes in these outcomes.

### 3.2. Perceived Stress

**Figure 2:**
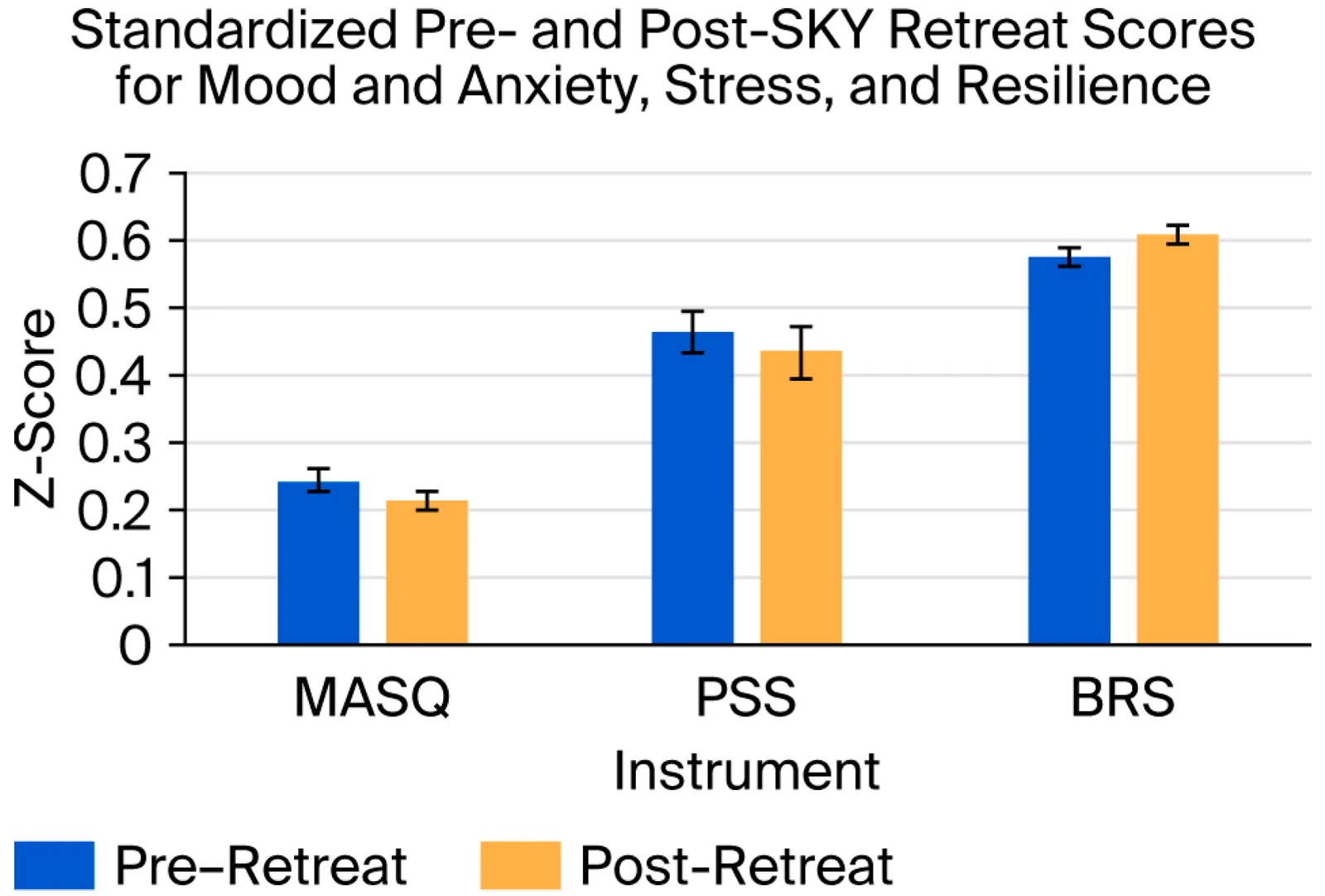
Preintervention and postintervention scores for mood and anxiety symptoms, perceived stress, and resilience. Error bars represent the standard error of the mean.

Mean PSS scores decreased from preintervention (M = 18.50, SD = 4.583) to postintervention (M = 17.30, SD = 5.841). This reduction was statistically significant, *t*(43) = 2.072, *p* = 0.022, 95% CI [0.032, 2.377], Cohen’s *d* = 0.312 (Figure 2).

Although the effect was small to moderate, the reduction suggests that the retreat may help students manage perceived academic and clinical stress. This finding is consistent with prior studies of SKY© and with broader evidence supporting mindfulness- and breath-based stress-management interventions among university and nursing students [14,15,16]. The relatively modest effect may reflect the retreat’s brief duration, variation in participant engagement, or the persistence of external academic demands following the intervention. Therefore, the finding should be interpreted as preliminary evidence of an association rather than definitive evidence of effectiveness.

### 3.3. Resilience

Mean BRS scores increased from preintervention (M = 19.80, SD = 3.414) to postintervention (M = 20.61, SD = 3.499). This improvement was statistically significant, *t*(43) = −2.501, *p* = 0.008, 95% CI [−1.478, −0.158], Cohen’s *d* = −0.377, representing a small-to-moderate effect (Figure 2).

The improvement in resilience suggests that participants perceived a modest increase in their ability to recover from stressful experiences. Breathwork, mindfulness, reflection, and community-building activities may support adaptive coping and emotional self-regulation, which are conceptually related to resilience. Nevertheless, resilience is a multidimensional capacity that generally develops over time. The relatively small change observed after a three-day retreat should therefore be interpreted cautiously. Longitudinal research is needed to determine whether this improvement persists and whether continued practice strengthens the effect.

### 3.4. Participant Perceptions

Participants rated the overall quality of the retreat an average of 3.58 on a five-point scale. Among the 42 participants who responded to the recommendation item, the mean likelihood of recommending the retreat to friends or colleagues was 5.85 on a 10-point scale.

These ratings indicate moderate rather than uniformly strong acceptance of the retreat. The participant perceptions provide an important contrast to the statistically significant changes in stress, distress, and resilience: measurable improvement did not necessarily translate into strong satisfaction or willingness to recommend the experience. This difference may reflect individual preferences, the intensive retreat format, scheduling demands, or aspects of implementation; however, these possible explanations were not directly assessed.

### 3.5. Implications for Nursing Education

Collectively, the findings suggest that the SKY Happiness Retreat© may hold promise as a wellness-focused experiential activity for prelicensure nursing students, particularly for addressing perceived stress and general distress. Structured wellness interventions may provide students with strategies for supporting personal well-being while navigating demanding academic and clinical environments. The intervention’s emphasis on resilience, self-awareness, and adaptive coping conceptually aligns with areas emphasized in the AACN Essentials (2021), including personal well-being and professional identity formation.

However, this study did not assess nursing competencies, course learning outcomes, or implementation indicators and therefore cannot establish the retreat as an appropriate clinical experience for mental health nursing education. The single-group design also prevents attribution of the observed changes specifically to the retreat. Larger controlled studies involving more diverse nursing student populations, longitudinal follow-up, and formal assessments of acceptability and implementation are needed before curricular or clinical adoption can be recommended.

## 4. Limitations

Several limitations should be considered when interpreting these findings. The small sample size, recruitment from a single rural institution, and predominantly White female sample limit the generalizability of the results to nursing students from other institutions, geographic settings, and more diverse demographic backgrounds. The limited sample size also reduced the study’s ability to detect broader patterns and increased the potential influence of individual variation on the results.

Another major limitation is the absence of a control group, making it difficult to determine whether the observed reductions in stress and improvements in resilience were directly attributable to the SKY Happiness Retreat© or to other factors. Without a comparison group, it is not possible to rule out alternative explanations such as natural fluctuations in stress over time, maturation effects, or placebo effects.

Additionally, threats to validity must be acknowledged. Participants’ age, personal circumstances, and previous life experiences may have influenced their stress levels and resilience-building capacity, introducing variability not controlled for in the study design. Furthermore, the timing of the retreat during the mid-spring semester may have affected student responses, as academic workload, clinical expectations, and external stressors fluctuate throughout the semester.

Another limitation is the reliance on self-reported measures, including the Perceived Stress Scale (PSS), Brief Resilience Scale (BRS), and Mini-MASQ. These instruments assess participants’ perceptions of stress and resilience at a specific point in time rather than objectively measuring long-term behavioral or psychological changes. While significant improvements were observed in generalized distress, the students’ long-term ability to manage anxiety arousal and anhedonic depression requires additional follow-up to determine whether the benefits were sustained over time.

Interestingly, participants rated the retreat only moderately favorably and expressed limited willingness to recommend it. This discrepancy suggests that measurable psychological outcomes and participant acceptability represent distinct dimensions of an intervention. Students may experience some benefit without perceiving the format as enjoyable, convenient, or appropriate for curricular integration. Factors such as scheduling demands, the intensive three-day format, unfamiliarity with breathwork practices, or discomfort participating in group mindfulness exercises may have influenced participants’ perceptions; however, these factors were not directly assessed and remain speculative. Because additional qualitative or implementation data were not collected, the reasons for the moderate ratings cannot be determined. Future studies should incorporate qualitative feedback and validated measures of acceptability, feasibility, intervention fidelity, and sustained engagement before recommending the retreat for broader integration into nursing curricula.

As a pilot investigation, the primary purpose of this study was to evaluate feasibility and generate preliminary evidence rather than establish definitive effectiveness. Although statistically significant improvements were observed in several outcomes, these findings should be interpreted as preliminary evidence supporting the feasibility and potential effectiveness of the intervention until replicated in larger, adequately powered randomized studies.

## 5. Recommendations for Future Research

Future studies should employ randomized controlled designs with larger and more diverse samples to strengthen causal inference and improve generalizability. Longitudinal follow-up is needed to determine whether improvements in stress and resilience persist throughout nursing education and into professional practice. Studies should include qualitative feedback and formal measures of acceptability, feasibility, participation, and intervention fidelity to clarify how students experience the program and identify opportunities for improvement.

Research should also evaluate alternative methods of delivering the intervention. Hybrid or virtual formats supplemented with periodic reinforcement sessions may improve accessibility and participant engagement while allowing students to integrate stress management techniques throughout an academic semester. Comparing different delivery formats may identify approaches that maximize both effectiveness and student satisfaction.

Finally, additional research should examine whether intervention effectiveness differs according to demographic characteristics, previous mindfulness experience, baseline mental health status, or stage within the nursing curriculum. Identifying which students derive the greatest benefit will help educators design targeted wellness initiatives that promote resilience across diverse nursing student populations.

Given the demanding nature of nursing, it is essential for future nurses to develop effective strategies for managing stress, particularly anxious arousal and generalized distress, as these factors can significantly impact clinical performance. Nurses who struggle with managing stress, especially in high-pressure environments such as acute care settings, may find it challenging to provide optimal patient care. The ability to remain composed and resilient in stressful situations is critical for ensuring both personal well-being and patient safety.

Future research should explore the contextual factors that influence stress and resilience in nursing students and professionals, including job roles, work environments, and individual differences. Understanding these factors can help develop more targeted interventions that foster emotional intelligence, self-efficacy, and resilience, ultimately benefiting both nurses and their patients.

Additionally, integrating mindfulness practices and technological tools into nursing education and clinical settings may offer effective strategies for stress management. Programs designed to enhance resilience should focus on immediate stress reduction and long-term mental well-being to ensure nurses can navigate the emotional and physical demands of their profession. Longitudinal studies assessing the sustained impact of such interventions will be valuable in guiding the development of comprehensive support systems for nursing professionals.

## 6. Conclusions

The findings of this exploratory pilot study suggest that participation in the SKY Happiness Retreat© may be associated with reduced perceived stress and general psychological distress and modestly improved resilience among nursing students. No statistically significant changes were observed in anxious arousal or anhedonic depression, suggesting that a brief retreat-based intervention may have limited influence on more persistent symptoms of anxiety and depression.

The SKY Happiness Retreat© may represent a promising wellness-focused experiential activity for prelicensure nursing students, including those enrolled in rural programs with limited access to specialty mental health clinical experiences. However, these preliminary findings should not be interpreted as evidence of definitive effectiveness or broad curricular applicability. The small convenience sample, single-group pretest–posttest design, absence of a control group, reliance on self-reported measures, and recruitment from a single rural institution with a predominantly White female sample limit causal inference and generalizability. Larger controlled studies involving more diverse nursing student populations, longitudinal follow-up, and formal assessments of feasibility and acceptability are needed to determine the intervention’s effectiveness, sustainability, and potential role in nursing education.

## Data Availability

The data supporting the findings of this study are available from the corresponding author upon reasonable request, subject to applicable ethical and privacy restrictions. Neither the Art of Living Foundation nor the International Association for Human Values has access to or control over the study data.

## Public Involvement Statement

No public involvement in any aspect of this research.

## Guidelines and Standards Statement

This manuscript was prepared in accordance with the Transparent Reporting of Evaluations with Nonrandomized Designs (TREND) Statement for reporting nonrandomized intervention studies.

## Use of Artificial Intelligence

ChatGPT (OpenAI; https://chatgpt.com; accessed on 24 June 2026) and Grammarly (https://www.grammarly.com; accessed on 24 June 2026) were used to assist with editing, improving grammar and readability, and enhancing the clarity and organization of the manuscript. The authors reviewed, revised, and verified all content and take full responsibility for the accuracy, integrity, and originality of the work. No AI tools were used to generate, analyze, or interpret the study data or to make scientific conclusions.

## Abbreviations

The following abbreviations are used in this manuscript:

BRS	Brief Resilience Scale
BSN	Bachelor of Science in Nursing
CI	Confidence Interval
EI	Emotional Intelligence
M	Mean
MBSR	Mindfulness-Based Stress Reduction
Mini-MASQ	Mini Mood and Anxiety Symptom Questionnaire
PSS	Perceived Stress Scale
QoL	Quality of Life
SD	Standard Deviation
SKY©	SKY Happiness Retreat©
